# Oral supplement in healthy older adults to support physical fitness and mental wellbeing

**DOI:** 10.3389/fnut.2025.1563999

**Published:** 2025-05-12

**Authors:** Amanda J. Lloyd, Robert J. Nash, Alina Warren-Walker, Alison Watson, MJ Pilar Martinez Martin, Courtney Davies, Bernardo Villarreal-Ramos, Thomas Wilson, Manfred Beckmann

**Affiliations:** ^1^Department of Life Sciences, Aberystwyth University, Aberystwyth, United Kingdom; ^2^Phytoquest Limited, Institute of Biological, Environmental and Rural Sciences (IBERs), Aberystwyth, United Kingdom

**Keywords:** iminosugar, Pittsburgh Sleep Quality Index, Q-actin, flow infusion electrospray ionization mass spectrometry (FIE-MS), melatonin derivatives

## Abstract

**Introduction:**

Cucumbers have been anecdotally credited with anti-inflammatory properties. IdoBR1 [(2*R*,3*R*,4*R*,5*S*)-3,4,5-trihydroxypiperidine-2-carboxylic acid], an iminosugar amino acid isolated from the fruits of certain cucumbers (*Cucumis sativus, Cucurbitaceae*), has been demonstrated to possess anti-inflammatory activity. Q-actin™ is a quality-controlled cucumber extract containing measured idoBR1, which has shown promising results in the treatment of osteoarthritis through oral administration.

**Methods:**

A healthy middle-aged and older adult population was recruited and randomized to receive either Q-actin™ (2 × gummies containing 10 mg Q-actin™ daily) or matched placebo gummies for 12 weeks. Physical strength and finger dexterity were assessed using hand grip strength and the Nine-Hole Peg Test, respectively. We evaluated diet choices with the Prime Diet Quality Score and sleep quality with the Pittsburgh Sleep Quality Index and collected first-morning urine samples for chemical composition analysis using Flow Infusion Electrospray Ionization Mass Spectrometry (FIE-MS).

**Results:**

We recruited a cohort of 47 healthy middle-aged and older adults (ages 50–78; 15 men and 32 women). FIE-MS analysis on urines was conducted and we tentatively identified elevated melatonin derivatives after Q-actin™ supplementation, suggesting a positive effect on sleep quality, which correlated with self-reported Pittsburgh Sleep Quality Index. The relative *m/z*-transition areas of melatonin and its two main biotransformation products in selected urine samples after Q-actin™ supplementation were confirmed following LC-MS/MS fragmentation.

**Conclusion:**

Q-actin™ demonstrated potential benefits in a healthy middle-aged and older adult population by improving sleep quality, as evidenced by elevated melatonin derivatives identified in urine samples and self-reported improvements on the Pittsburgh Sleep Quality Index and improved finger dexterity. These findings support the hypothesis that Q-actin™ may have positive effects on overall wellbeing, as shown here in healthy older people, and could be rated to enhanced sleep quality.

**Clinical trial registration:**

ISRCTN registry ISRCTN28705061 and clinicaltrials.gov/ NCT05878847.

## 1 Introduction

Cucumbers have been anecdotally credited with anti-inflammatory properties, though the specific active ingredient was identified only recently (1) idoBR1 [(2*R*,3*R*,4*R*,5*S*)-3,4,5-trihydroxypiperidine-2-carboxylic acid], is an iminosugar amino acid isolated from the fruits of certain cucumbers (*Cucumis sativus, Cucurbitaceae*), that has been shown to possess anti-inflammatory activity (1, 2). IminoTech Inc., based in the USA, in collaboration with Phytoquest Ltd., which discovered idoBR1, developed a quality-controlled standardized cucumber extract containing over 1% idoBR1 content, called Q-actin™. This extract has shown promising results in the treatment of osteoarthritis through oral administration (3).

Chronic inflammation is increasingly recognized as a major contributing factor to age-related health decline, including cognitive deterioration and sleep disturbances (4). Inflammatory cytokines such as interleukin-6 (IL-6) and tumor necrosis factor-alpha (TNF-α) have been implicated in the modulation of sleep patterns, with elevated levels associated with impaired sleep quality and duration (5). The potential anti-inflammatory effects of idoBR1 could, therefore, provide a novel therapeutic strategy for improving sleep and cognitive function in aging populations.

Iminosugars are primarily recognized for their roles as glycosidase inhibitors, with therapeutic applications in conditions like diabetes, Gaucher disease, immunosuppressive activities, and antibacterial and antiviral effects (6). Several peer-reviewed studies have investigated the effects of idoBR1 and Q-actin™, particularly focusing on their potential benefits in managing osteoarthritis (3, 7). Some studies suggest that certain iminosugars may influence physiological processes that could indirectly affect sleep. A preclinical study demonstrated that idoBR1 reduced pro-inflammatory factors released by microglia—specialized immune cells in the brain. Poor sleep raises inflammatory markers (8) but inflammation can also impact sleep patterns and overall health (9).

Aging is associated with a decline in melatonin production, which can lead to sleep disturbances and increased oxidative stress (10). After its secretion, melatonin undergoes hepatic metabolism, primarily being hydroxylated to 6-hydroxymelatonin, which is subsequently conjugated to form 6-sulfatoxymelatonin (11). These metabolites are predominantly excreted in urine, with 6-sulfatoxymelatonin serving as a reliable biomarker for assessing endogenous melatonin production.

The quantification of these urinary metabolites offers a non-invasive method to monitor melatonin dynamics in clinical and research settings. While iminosugars have demonstrated various health benefits, direct evidence of their impact on sleep quality is currently limited. The present study aims to evaluate the effects of Q-actin™ supplementation on physical performance, sleep quality, and overall wellbeing in healthy middle-aged and older adults. By using non-invasive urinary metabolomics techniques such as FIE-MS, we aim to explore potential biochemical mechanisms underlying the observed effects of Q-actin™ on sleep and cognitive function.

## 2 Material and methods

### 2.1 Subjects

This was a double-blind, randomized, placebo controlled parallel human clinical trial [registered in the ISRCTN registry (https://www.isrctn.com/) under registry number ISRCTN28705061 and Clinical trials.gov under registry number (clinicaltrials.gov/) NCT05878847], approved by the Research Ethics Panel at Aberystwyth University (23767). Subjects were recruited by the Wellbeing and Health Assessment Research Unit (WARU) at Aberystwyth University and the study was carried out in accordance with the Declaration of Helsinki, where participants gave written informed consent. The participants were healthy and not selected for sleep or anxiety problems (see eligibility criteria). The pre-screening for the first volunteers started in December 2022 and the study was completed in April 2023. The trial was conducted at Canolfan Plas Y Sarn (Leisure Center), Trimsaran and WARU and the participants followed the intervention trial at home and came to one of the two venues at week 0 (baseline), at 6 weeks and at the end of the trial (12 weeks) for measurements, tasks, urine collections, and questionaries.

Participants were recruited according to eligibility criteria. Inclusion criteria included: consenting adults >50 years of age; commit to urine sampling; able to commit to attending Canolfan Plas Y Sarn or WARU for measurements of physical strength, finger dexterity, diet choices, and sleep; able to restrict from consumption of cucumber, gherkins, and melon for 2 days before coming for measurements. Exclusion criteria included: serious health conditions that require daily long-term medications (including immunosuppressants); a history of current diabetes, lung issues, gut inflammation (Crohn's, IBD), digestive disorders; diagnosed with a serious health condition within the last 12 months; pregnant or lactating; play sports at a high level (more than 7 h/week or 1 h/day); smoker; consume high dose of alcohol >21 unit per week for men and >14 units per week for women; food allergy/food intolerance/eating disorder or are on a specially prescribed diet. If eligible, participants were allocated to two groups using computer-generated random number tables: Q-actin™ or placebo gummy. The study was double-blinded, so researchers and intervention participants were unaware of the allocation until intervention and analysis completion.

### 2.2 Sample size calculation

There were multiple minimum differences (small, medium and large) between Q-actin and placebo that were considered clinically meaningful for the primary and secondary outcomes (e.g., self-report, grip strength, Nine-Hole Peg Test, etc.) due to the experimental mixed model design. Additional, high-throughput metabolomics is about hypothesis generation, rather than looking for clinically meaningful outcomes. Therefore, we adjusted the power calculation for repeated measures and moderate-large effect size (d = 0.5–0.6), so the required sample size is reduced to approximately 22–32 participants per group (45–64 total), due to the design effect from repeated measurements (assumed correlation r = 0.5).

### 2.3 Study design: dose and type

The study was split into three experimental sessions (0, 6, and 12 weeks) where the participant was to be randomized to groups; group one would be given a daily dose of two Q-actin™ gummies daily, and group two would be given two placebo gummies daily, matched by taste and appearance to Q-actin™ gummies. Gummies were supplied for a period of 12 weeks. The gummies (ingredients in Appendix 1) were to be consumed in the evening, before bedtime.

### 2.4 Anthropometric measures

Body weight (using SECA 799 Electronic Column Scales to the nearest 0.1 kg) and height was measured (using a Holtain Stadiometer to the nearest cm) to allow the body mass index (BMI) to be calculated and compared to ranges set out by the WHO (12). Waist circumference (midway between the lower rib and the iliac crest on the midaxillary line) and hip (level of the widest circumference over the great trochanters) were taken to the nearest 0.1 cm, using an ergonomic circumference measuring tape over bare skin, whenever possible. Triplicate measurements were made, and the mean was calculated, allowing the waist-to-hip ratio to be calculated and compared to published ranges (13, 14). All measurements were done in the morning, by the researcher after the participants had been fasting for at least 8 h.

### 2.5 Testing and self report

The participants came to the Centers for physical strength and finger dexterity measurements using hand grip strength (Grip Strength Dynamometer T.K.K. 5401, Takei Scientific Instruments, Japan) and the Nine-Hole Peg Test (9HPT) respectively (Appendix 2).

Diet was assessed using Prime Diet Quality Score (PDQS) (15), amended to account for vegetarian and vegan choices which assessed an individual's diet and eating habits. Sleeping habits were recorded using the Pittsburgh Sleep Quality Index (PSQI) (16).

Participants filled in self-reporting questionnaires with no prompting from researchers on the 1^st^ day of the trial, at 6 weeks and at 12 weeks. All participants were requested to maintain their physical activity routine. To evaluate compliance, participants were requested to note any missed gummies and to bring back the remaining gummies.

Mixed-model ANOVA were conducted using SPSS V27 (IBM corporation) to determine relational effects between Q-actin supplementation on finger dexterity, physical strength, sleep quality, and diet. The within-subject factor was study time-point, the between- subject factor was supplement type, and the dependent variables were grip strength score (for physical strength), peg-test speed (for finger dexterity), PSQI total sleep score (for sleep quality), and PDQS score (for diet quality). Data were checked for normality using the Shapiro-Wilk test (*p* > 0.05). For finger dexterity and grip strength, two mixed-model 3 (timepoint) × 2 (supplement type) ANOVAs (for 29 full datasets at all three time-points) for finger dexterity and grip strength, were used to test the null hypothesis that: participants consuming Q-actin for a 12-week period would have improved finger dexterity (measured as a reduction in time taken to complete 9-hole peg test) and grip strength (measured as an increase in grip strength score) compared to those participants taking the placebo control. For the self-report 5 mixed model 3 (timepoint) × 2 (supplement type) ANOVAs (for 29 full datasets at all three time-points) for diet score, diet percentage, and the PSQI, were used to test the prediction that: participants consuming Q-actin for a 12-week period would have better sleep quality (decreased PSQI total sleep score) and better diet (increased PDQS score and percentage), compared to the placebo control.

### 2.6 Collection and preparation of urine samples and analysis

Each participant was requested to collect duplicate first morning void (FMV) urine samples at home using the vacuum transfer system as described in Lloyd et al. (17). Written instructions and a video on how to collect FMV urine samples were provided. All samples were stored at home at 4°C and then brought to the Center. At the Center, tubes which were centrifuged at 3,000 x*g* for 5 min; the contents of one tube was transferred to a clean four-ml cryotube, which was labeled and stored at −80°C; the contents of the second tube was aliquoted in a series of 2 mL Eppendorf tubes (typically 3 × 1 mL or 6 × 0.5 mL aliquots). Once aliquoted, the samples were stored at −80°C, allowing single aliquots to be removed when needed for analysis. The remaining supernatant in the vacutainer was then used for Refractive Index (RI) measurements.

### 2.7 Processing of urine samples

Urine samples were prepared and adjusted as reported previously (18–20). In brief, all urine samples were normalized by refractive index (RI) prior to analysis to account for differences in fluid intake by participants and to ensure that all Mass Spectrometry (MS) measurements were made within a similar dynamic range within the linear range of the instrument. Samples were defrosted overnight at 4°C, centrifuged (1,600 × g for 5 mins at 4°C), placed on ice and aliquots of thawed urine (1,000 μL) were transferred into labeled 2 mL Eppendorf tubes. The remaining sample were returned to a −20°C freezer. An OPTI Handheld Refractometer (Bellingham Stanley™ Brix 54 Model) was used to record the specific gravity (SG). Aliquots of the required amounts of urine from centrifuged 2 ml Eppendorf tubes were adjusted to the same SG with ultra-pure (18.2 Ω) H_2_O. Adjusted urine samples were extracted with an equal volume of 100% methanol.

### 2.8 Flow infusion electrospray ionization mass spectrometry analysis

FIE-MS analysis was conducted with an Exploris 120 mass analyser coupled with a Dionex Vanquish ultra high performance liquid chromatography (UHPLC) system (Thermo-Scientific), measuring ion intensities within the m/z range of 55 to 1,200. The mobile phase was methanol:water (70:30). Metabolite fingerprints were generated in both positive and negative ionization modes, in a single run. The data obtained was subjected to spectral binning followed by multivariate analysis (21).

All samples were randomized to minimize batch effects. Samples (20 μl) were injected into a flow of 100 μl min^−1^ methanol:water (70:30, v/v). Ion intensities were acquired between *m/z* 55 and 1,200 for 3.5 min at a resolution setting of 120,000, resulting in 3 (±1) ppm mass accuracy. Tuning and ESI source parameters were set according to manufacturer's recommendations. Following data acquisition, Chromeleon.cmbx files were first exported to .raw files and then converted to the .mzML open file format and centroided (22) using msconvert (TransProteomicPipeline) (23). Spectral binning was applied using the R package binneR (24) and then standard post-acquisition processing routines were applied, including occupancy and QC filtering. Putative Molecular formulas were generated by using MZedDB (25), an Aberystwyth University database for accurate mass annotation.

Intra-batch variance was removed by adjusting intensity values by the mean value of the respective analytical block (where each block contains an equal representation of the total biological variance). Prior to statistical analysis, data were normalized to the total ion count (TIC) of the sample. For multivariate analysis, all samples and features were used.

Principal Component Analysis (PCA) was followed by PC-Linear Discriminant Analysis (PC-LDA). PCA was performed using the *prcomp* function in R (26). Supervised Random Forest (RF) classification was implemented using the randomForest package in R (26). For all Random Forest models, the number of trees (*ntree)* used was 1,000 and the number of variables considered at each internal node (mtry) was the square root of the total number of variables. RF classification models were plotted following multi-dimensional scaling (MDS). Proximity measures for each individual observation were extracted from RF models and scaled coordinates produced using *cmdscale* on 1 – proximity.

Top ranked features contributing to the MDS models were extracted using re-sampling methods and *p*-values of False Positive rates (FPR ≤ 0.05).

### 2.9 Targeted liquid chromatography-tandem mass spectrometry

Standards of melatonin, 6-hydroxymelatonin, and 6-sulfatoxymelatonin were purchased from LGC Standards, Canada, for precise and reliable analysis. The quantification of these compounds was conducted using a Vanquish ultra performance (UP) LC system coupled with a TSQ Quantis mass spectrometer (Thermo Scientific). Chromatographic separation was achieved with a Hypersil Gold reversed-phase column (2.1 mm × 200 mm, 1.9 μm; Thermo Scientific). The analysis utilized multiple reaction monitoring (MRM) mode to ensure high sensitivity and specificity, with the most intense ion transitions monitored as follows: melatonin (*p*233.0 → *p*174.1), 6-sulfatoxymelatonin (*n*327.1 → *n*247.1), and 6-hydroxymelatonin (*p*249.1 → *p*190.1). This setup provided a robust and reliable method for the detection and quantification of the analytes.

## 3 Results

### 3.1 Overall compliance

**Figure 1:**
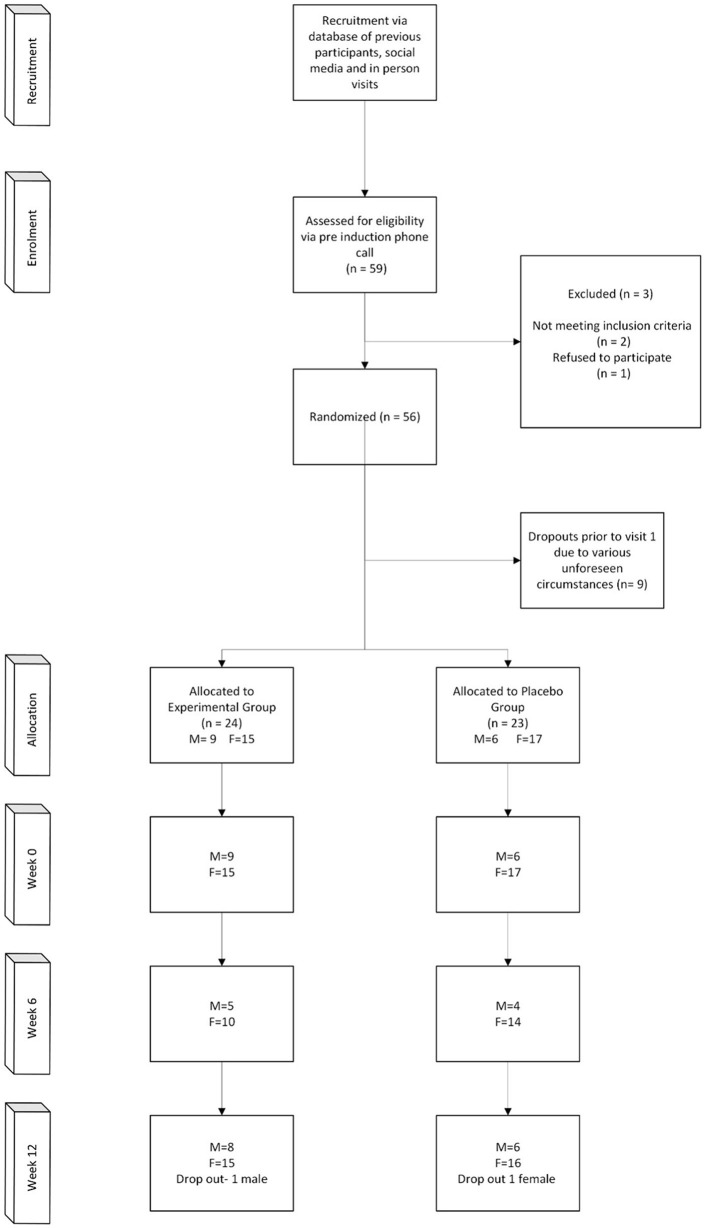
CONSORT diagram showing the flow of participants through each stage of the randomized trial.

A total of 56 participants were randomized, however only 47 participants started the trial due to various reasons (Figure 1). The power calculation for repeated measures, was approximately 22–32 participants per group (45–64 total), the sample size of 47 participants was powered for a large effect and assumed correlation among repeated measures. The mean age and BMI of the participants were 63.03 ± 7.13 (years) and 29.94 ± 4.85 (kg/m^2^), respectively (Table 1). Not all participants provided samples and self-report data at 6 weeks, however at 12 weeks all participants provided samples and self-report data, except for two participants that did not attend. Based on the residual gummies in the packed sachets, participants had high adherence and compliance rate was over 99%, and no serious adverse events were associated with the trial. Two people reported to take sertraline, one from the intervention group and one from the placebo group. No other benzodiazepines, antidepressant and anti-histaminic drugs were taken during the study period.

**Table 1:** Characteristics of the study participants at baseline.

**Q-Actin**			**Placebo**			**P-value**
**Variable**	**Mean**	**(SD)**	**Variable**	**Mean**	**(SD)**	
**Demographics**			**Demographics**			
Total [n]	24		Total [n]	23		
Gender female [n]	15		Gender Female [n]	17		0.412
Gender male [n]	9		Gender Male [n]	6		
Age [years]	64.14	6.82	Age [years]	64.41	6.7	0.894
Age range (min-max) [years]	50–77		Age range (min-max) [years]	52–74		
**Anthropometrics**			**Anthropometrics**			
Weight [kg]	80.36	12	Weight [kg]	89.17	17.3	0.048
Height [cm]	167.7	9.18	Height [cm]	168.6	9.31	0.745
BMI [kg m^−2^]	28.7	4.67	BMI [kg m^−2^]	31.29	4.96	0.075
Waist circumference [cm]	99.57	10.9	Waist circumference [cm]	102.6	19	0.506
Hip circumference [cm]	105.1	12.4	Hip circumference [cm]	111.1	17.4	0.203
Waist to Hip ratio	0.95	0.14	Waist to Hip ratio	0.92	0.08	0.325

Data are presented as mean (SD) for continuous variables and as number of participants or percentage (%) for categorical variables. Body Mass Index (BMI) was calculated as [weight(kg)/height(m)^2^]. Independent samples *T*-test for baseline demographic comparisons between Q-Actin and Placebo Groups conducted.

### 3.2 Self-report

**Figure 2:**
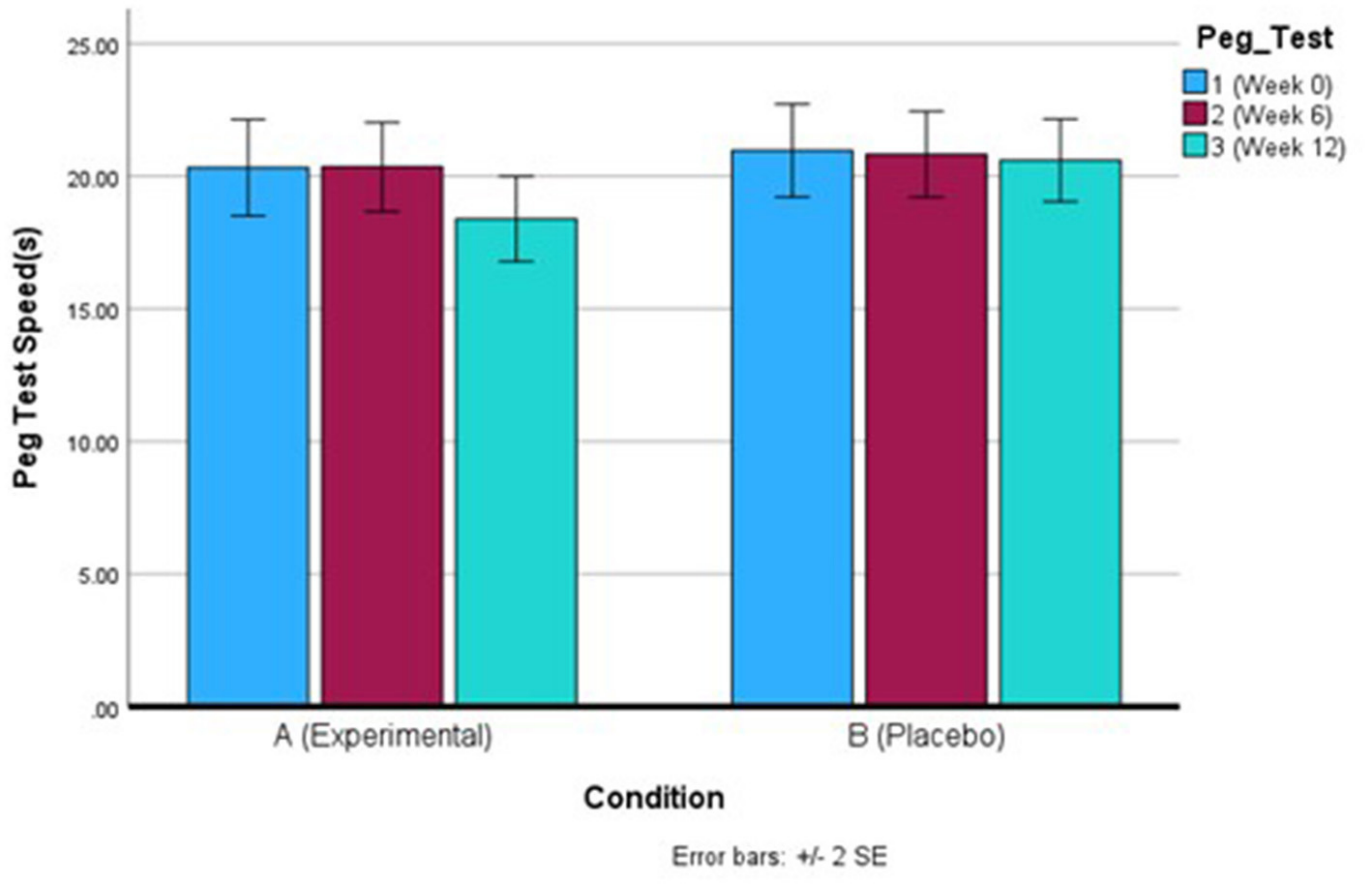
Nine-Hole Peg Test (9HPT) speed for the Q-actin™ group **(A)** and placebo **(B)** for weeks 0, 6, and 12.

For grip strength participants in both groups showed a significant improvement between week 0 and week 6, with no change in strength at week 12. There were no significant differences in strength between the two groups, suggesting both Q-actin™ and placebo both improved average grip strength following consumption (Table 2). For dexterity, in the Q-actin™ group, participants showed a significant improvement between week 6 and week 12, achieving a faster peg test score on average. In comparison, participants in the placebo group showed no significant changes between weeks 0, 6, and 12, suggesting that consumption of Q-actin™ for 12 weeks significantly improved dexterity (Table 2, Figure 2).

**Table 2:** Statistical analysis of self-report questionnaires and tasks.

**Measure**	**Time-points**	**Q-actin™**	**Placebo**
Grip strength	Week 0 vs. 6	*p = 0.002*	*p = 0.006*
	Week 0 vs. 12	*p >* 0.05	*p >* 0.05
	Week 6 vs. 12	*p >* 0.05	*p >* 0.05
Peg test	Week 0 vs. 6	*p >* 0.05	*p >* 0.05
	Week 0 vs. 12	*p = 0.011*	*p >* 0.05
	Week 6 vs. 12	*p = 0.007*	*p >* 0.05
Diet percentage score	Week 0 vs. 6	*p >* 0.05	*p >* 0.05
	Week 0 vs. 12	*p >* 0.05	*p >* 0.05
	Week 6 vs. 12	*p >* 0.05	*p >* 0.05
PSQI	Week 0 vs. 6	*p >* 0.05	*p >* 0.05
	Week 0 vs. 12	*p >* 0.05	*p >* 0.05
	Week 6 vs. 12	*p >* 0.05	*p >* 0.05

Where: Effect size $\eta_{p}^{2}$:Grip Strength 0.320, Peg test 0.161.

For the PDQS, non-significant findings within-and-between groups were obtained for diet percentage scores across the 12-week intervention. This suggests that consumption of Q-actin™ or placebo for 12 weeks did not alter diet habits or lifestyle (Table 2).

**Figure 3:**
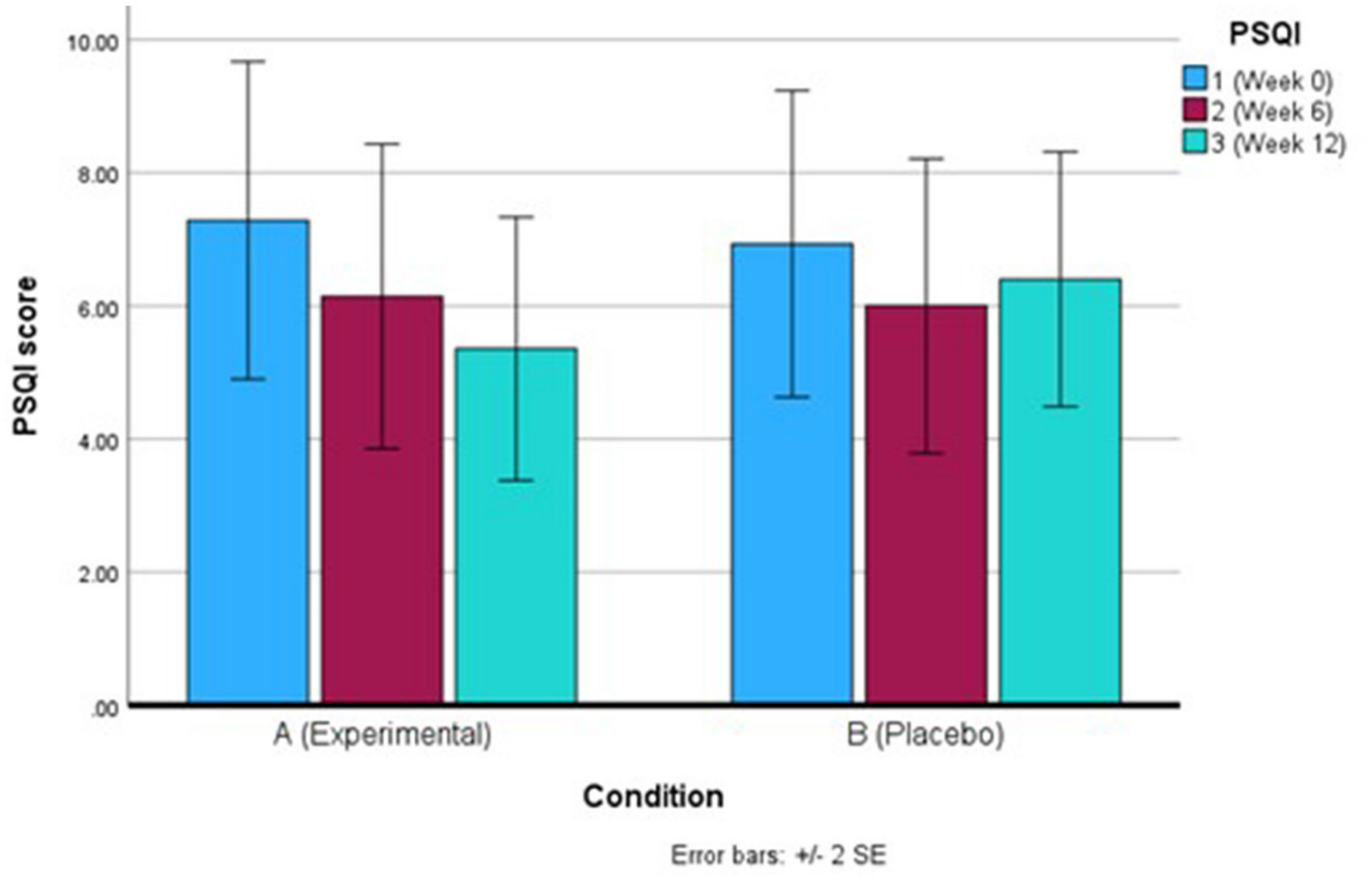
Mean Pittsburgh Sleep Quality Index (PSQI) score for the Q-actin™ group **(A)** and placebo **(B)** for weeks 0, 6, and 12.

For PSQI, after correcting for Greenhouse-Geisser following a violation of sphericity, there was a non-significant finding for total PSQI score both within-and-between groups. However, the decreasing PSQI score over the 12 weeks for the Q-actin™ group suggests there was an improvement in sleep quality overall (Table 2, Figure 3). By looking at component 1 (subjective sleep quality) only, there was a significant difference between weeks 0 and 6 (*p* = 0.020) for the Q-actin™ group, while the other components did not show any significant changes (data not shown).

### 3.3 FIE-MS analysis of urine

**Figure 4:**
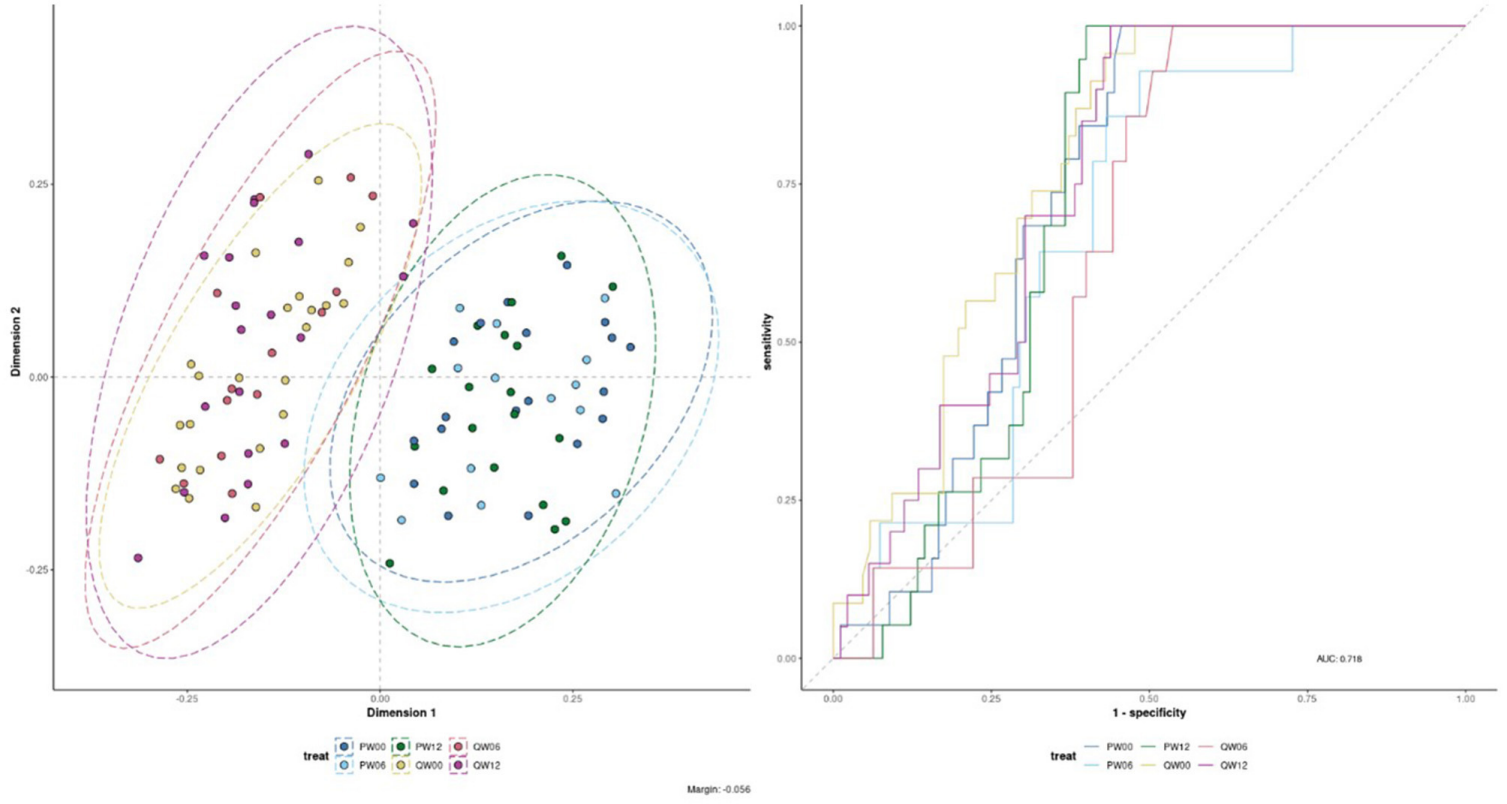
Multi-dimensional scaling (MDS) of Random Forest (RF) proximity values of the Flow Infusion Electrospray Ionization Mass Spectrometry (FIE-MS) fingerprint data. Where: Treatment: P, placebo; Q, Q-actin™; Week: 0 weeks, W00; 6 weeks, W06; 12 weeks, W12.

**Figure 5:**
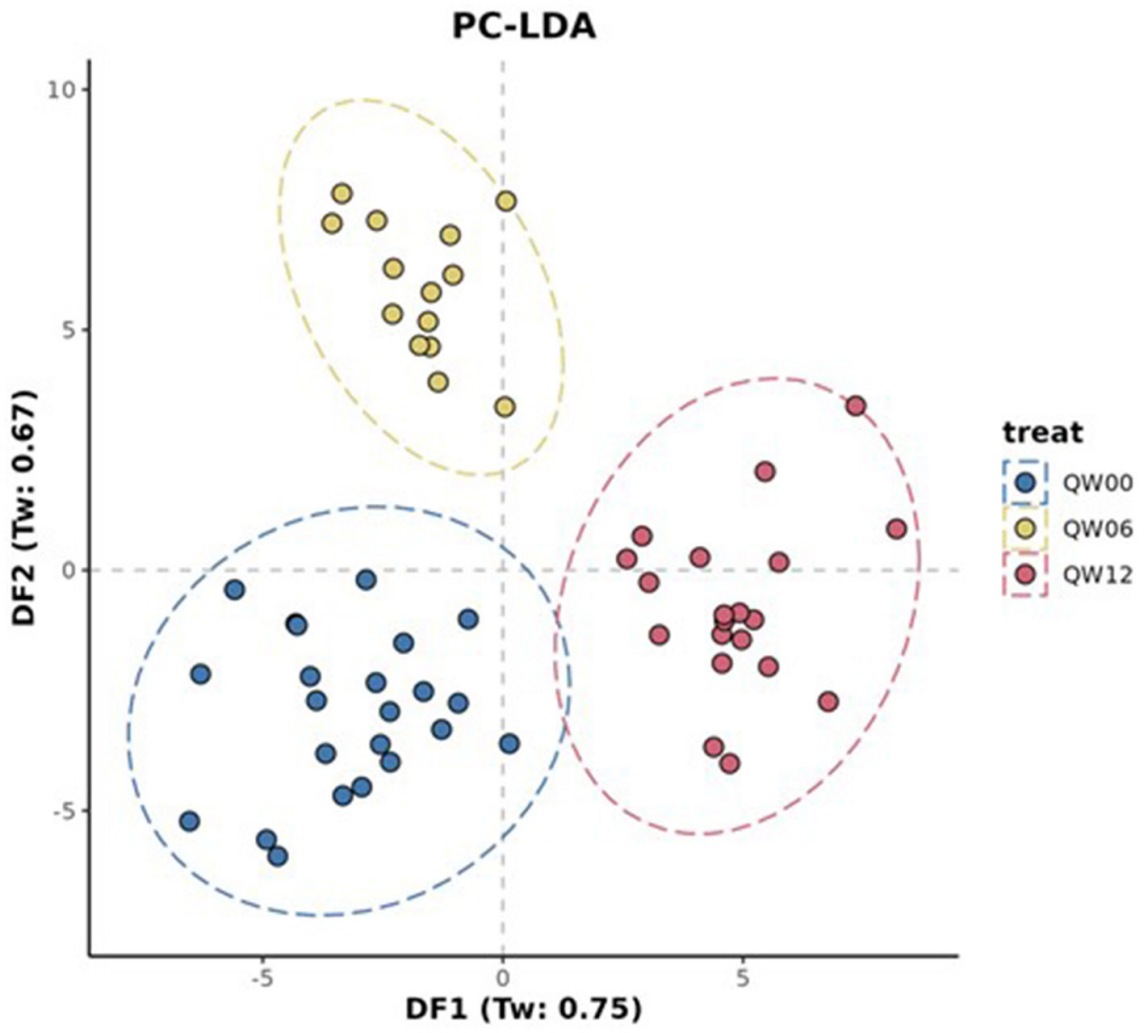
Principle component- Linear Discriminant Analysis (PC-LDA) of FIE-MS fingerprint data. Where: Treatment: Q, Q-actin™; Week: 0 weeks, W00; 6 weeks, W06; 12 weeks, W12. An ellipse around a population indicates the 95 % confidence interval.

**Figure 6:**
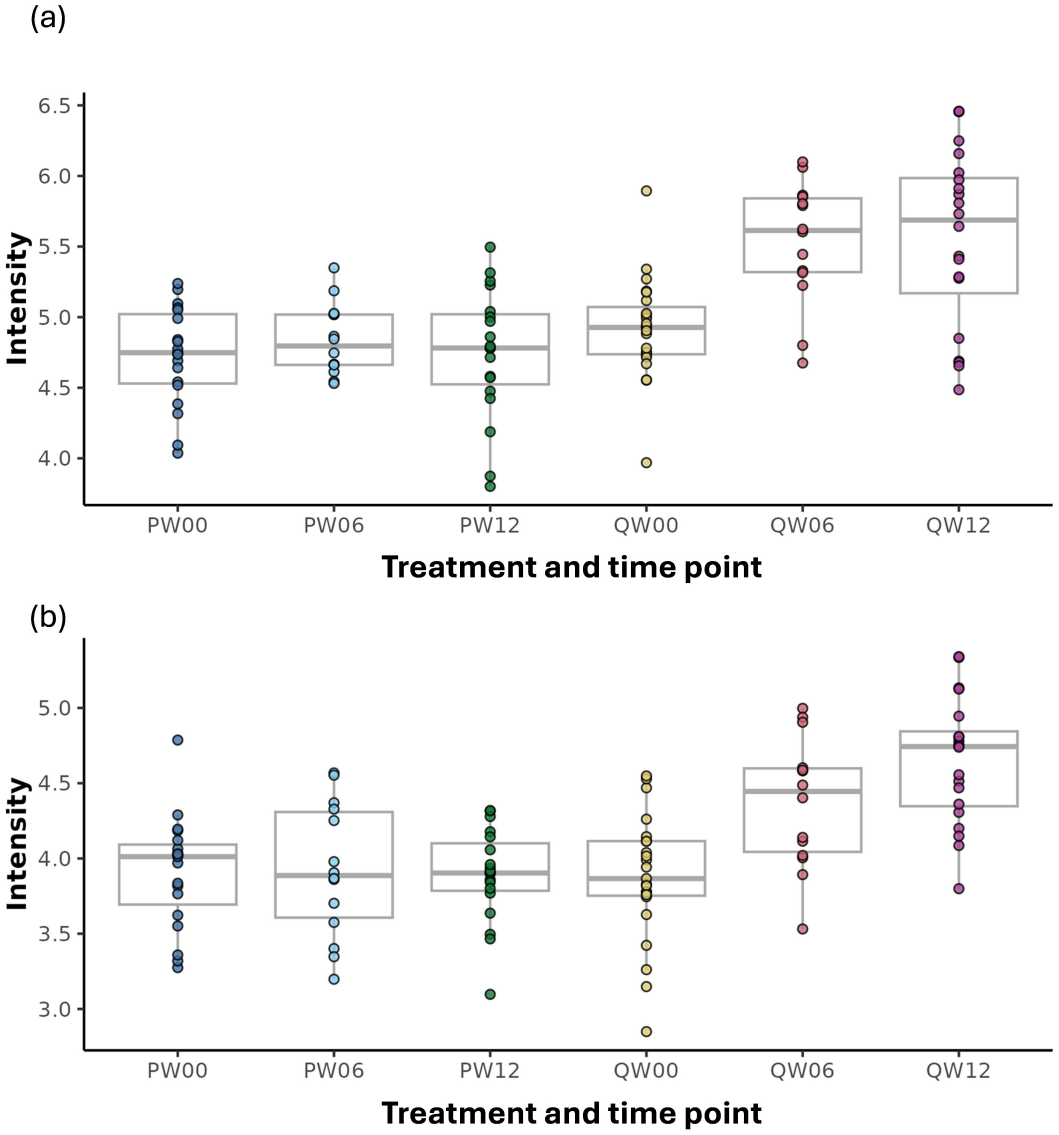
Box-plots of discriminatory features elevated after Q-actin™ supplementation compared with placebo control. Where **(a)** *m/z* n327.06564, tentatively Identified as sulfatoxymelatonin **(b)** *m/z* p271.10544 [M-H]^1−^, tentatively Identified as hydroxymelatonin [M+Na]^1+^. Where: Treatment: P, placebo; Q, Q-actin™; Week: 0 weeks, 00; 6 weeks, 06; 12 weeks, 12.

The processed FIE-MS fingerprint data contained 4,273 variables. RF classification models were plotted following MDS (Figure 4), showing separation of the Q-actin™ and placebo groups however no separation of the timepoints 0, 6, and 12 weeks (overlapping clusters observed). PC-LDA was used to visually separate the time points in each group (Q-actin™ group only, Figure 5). Despite very low margins, we explored the top ranked signals influencing the model. Two signals appeared highly ranked, these were sulfatoxymelatonin, annotated as [M-H]^1−^, and hydroxymelatonin [M+Na]^1+^, which both showed an increase after Q-actin™ consumption (Figures 6a, b respectively).

### 3.4 LC-MS/MS of urines

Targeted analysis by LC-MS/MS using standards confirmed the identification 6-hydroxymelatonin and 6-sulfatoxymelatonin, and we also identified melatonin, in the urine samples, validating the trends identified from the global metabolomic fingerprinting (Table 3). In the placebo urine samples the melatonin, 6-hydroxymelatonin and 6-sulfatoxymelatonin were below the detection limits (data not shown).

**Table 3:** Average concentration of 6-sulfatomelatonin,6-hydroxymelatonin and melatonin in RI-adjusted Q-actin™ urine samples (ug/ml).

**Intervention week**	**Average concentration in RI-adjusted urine samples (ug/ml)**		
	**6-sulfatomelatonin**	**6-hydroxymelatonin**	**Melatonin**
0	0.009	~	~
6	48.819	0.074	0.391
12	142.480	0.252	1.690

Where: ~ below the detection limits, all placebo urines had 6-sulfatomelatonin,6-hydroxymelatonin and melatonin concentrations below the detection limits.

## 4 Discussion and conclusion

The results of this study suggest that Q-actin™ supplementation may provide benefits in improving sleep quality and finger dexterity in healthy older adults.

Cucumbers have long been recognized for their potential anti-inflammatory properties, and recent research has identified idoBR1, an iminosugar amino acid, as the active component responsible for these effects (1, 2). In this study, we evaluated the effects of Q-actin™, a standardized cucumber extract standardized to >1% idoBR1 content, on physical and mental wellbeing in a healthy middle-aged and older adult population.

Q-actin™ has been clinically studied for its potential benefits in enhancing joint function and mobility (7). Its effects on motor function were further evaluated using the 9HPT, a validated measure of finger dexterity. Participants in the Q-actin™ group demonstrated faster completion times in the 9HPT over the 12-week intervention compared to baseline and placebo, in line with the results seen previously (7).

Participants who consumed Q-actin™ over 12 weeks exhibited a trend toward improved sleep quality, as evidenced by elevated urinary melatonin metabolites and self-reported improvements on the PSQI. Multivariate analysis of FIE-MS urinary data identified elevated concentrations of melatonin metabolites—specifically in FMV urine samples—from the treatment group at weeks 6 and 12 when compared with baseline and placebo.

Targeted LC-MS/MS using standards confirmed the presence of melatonin, 6-hydroxymelatonin, and 6-sulfatoxymelatonin in urine samples, validating the trends identified through global metabolomic fingerprinting. The observed increase in urinary melatonin metabolites following supplementation indicates a potential mechanistic link between Q-actin™ consumption and enhanced sleep regulation. Previous research has highlighted the role of bioactive compounds in regulating circadian rhythms, with specific plant-derived extracts demonstrating sleep-promoting effects (27).

Furthermore, the observed improvements in finger dexterity using the 9HPT suggest that Q-actin™ may have broader implications for neuromuscular function, which is closely linked to cognitive health (28). Declining dexterity and motor function are early indicators of age-related cognitive impairment, and interventions targeting these domains may offer dual benefits for both physical and mental wellbeing. The improvements in grip strength observed in both groups may reflect a placebo effect or learning effect from repeated testing. This emphasizes the importance of using objective biomarkers alongside self-report and performance-based measures.

To improve sleep quality there are medications available such as benzodiazepines, anti-depressants, and antihistamines but due to adverse effects there is a trend toward alternative sleep aids such as natural and herbal therapies (29). Other food components such as glycine, theanine, and gamma-amino butyric acid (GABA) have been said to promote sleep but their potential applications are limited. For example, the effective timing of glycine intake is limited to before bedtime and its effective dose which is in the order of several grams; GABA has little brain penetration and the effective dose of theanine is several 100 milligrams, which is slightly lower than that of glycine (30). The same authors report that a mushroom sulfur-containing amino acid, ergothioneine, has strong antioxidant and anti-inflammatory effects and can improve sleep in a clinical trial of people with high anxiety and sleep complaints. They also report that ergothioneine inhibits histamine N-methyltransferase and aldehyde dehydrogenase activity and binding to α3β4 nicotinic acetylcholine receptor and this might be related to the reported benefits to sleep. The common flavonoid luteolin is reported to have a hypnotic effect via Adenosine A1 and A2A Receptors (31), other flavonoids are also reported to have hypnotic effects but these are common in foods and so their contribution in the diet to sleep is not clear. In our study, this is the first iminosugar to be shown to have a potential effect on sleep. It is also notable that such a low daily dose (20 mg) of the cucumber extract seems effective. Although subjective improvements in sleep quality were modest, sleep disturbances are a known risk factor for cognitive decline, mood disorders, and reduced physical health in older adults. Therefore, even small improvements could have meaningful clinical implications, particularly if sustained long-term.

The integration of metabolomics with machine learning, specifically RF classification, enabled us to identify chemical differences between control and intervention urines. These findings offer new hypotheses into how this oral supplement in healthy older adults to support physical fitness and mental wellbeing. This approach highlights the potential of machine learning tools in metabolomics for uncovering complex patterns in high-dimensional data. The approach used in this paper is robust for generating hypotheses and identifying broad trends, however annotations based solely on molecular formulas remain tentative until confirmed by standards.

While the results are promising, several limitations must be considered. The study sample size was relatively small, and larger, more diverse cohorts are needed to confirm these findings. Future research incorporating predictor analyses could better understand individual variability in response to supplementation. Additionally, self-reported measures of sleep quality, such as the PSQI, may be subject to bias, and future studies should incorporate objective sleep monitoring techniques such as actigraphy or polysomnography. A targeted analysis of a broader panel of sleep markers could be used to evaluate whether Q-actin™'s ability to increase nocturnal melatonin levels makes it a beneficial supplement for promoting sleep in older populations. Further studies are required to confirm these results and explore additional health benefits of Q-actin™ supplementation.

The potential anti-inflammatory properties of Q-actin™ also warrant further investigation, as chronic low-grade inflammation has been implicated in numerous age-related conditions, including cardiovascular disease, neurodegeneration, and metabolic disorders (32). By reducing systemic inflammation, Q-actin™ may confer additional long-term health benefits beyond sleep improvement. Overall, these findings highlight the potential benefits of Q-actin™ in improving sleep quality, with implications for overall wellbeing in apparently healthy older adults including potentially supporting cognitive function. At present, it is not possible to determine whether there is a causal relationship between improved quality of sleep was the reason for improved cognitive/reaction effect.

In conclusion, this study provides preliminary evidence that Q-actin™ supplementation may support sleep quality and neuromuscular function in healthy older adults. Future research should explore the broader applications of iminosugar supplementation in age-related health management, with a focus on long-term efficacy and safety. Integrating metabolomics approaches with targeted biomarker analysis will be crucial for elucidating the underlying biological mechanisms.

## Data Availability

The raw data supporting the conclusions of this article will be made available by the authors, without undue reservation.
