# Supplementary material for: Oral supplement in healthy older adults to support physical fitness and mental wellbeing
Source: Front Nutr. 2025 May 12;12:1563999. doi: 10.3389/fnut.2025.1563999 (PMC12104766; doi:10.3389/fnut.2025.1563999)
Supplement: Supplementary file 1 [file Table_1.docx]

**Appendix 1:** Gummy ingredients (Q-actin was omitted in the placebo)


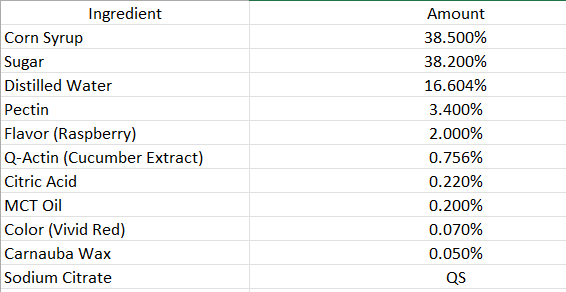


**Appendix 2:** Hand grip strength (hand held dynamometer) and Nine-Hole Peg Test (9HPT) SOPs

**Hand grip strength (hand held dynamometer)**

The test measurers the ability of the participant to squeeze a hand held dynamometer as tightly as possible

- 1. Provide the participant with the hand held dynamometer.
  2. Inquire about possible conditions that could affect the ability to complete the test, and record the dominant hand (ask: “*Are you right-handed, left-handed, or do you use both hands equally?*”.
  3. Adjust the grip size of the dynamometer until the second joint of the index finger is at a 90 degree angle on the handle (90˚ flexion between proximal and middle phalangeal joint). When adjusting the grip size, the hand should be in line with the wrist and forearm.
  4. Place the hand alongside the participant’s body, while the participant is standing upright and straight, with feet shoulder width apart.
     1. Note: if the participant is unable to stand, position the participant in a chair, while keeping the arm straight down during the attempt.
  5. Turn on the device and ensure the device calibrated.
  6. In their own time, the participant produces a maximal effort, squeezing the dynamometer as hard as possible for a few seconds.
     1. Pay attention whether the dynamometer doesn’t slip during the attempt; if it does, adjust the grip width based on the participant’s feedback.
  7. The dynamometer will register the maximal amount of force produced. Record the maximal amount of force.
  8. Reset the device. Repeat with the other hand. Repeat for both hands, ensuring a minimum of 15 seconds occurs between attempts for each hand.
  9. Record 3 attempts for each hand. The highest value is the outcome measure.

**Nine-Hole Peg Test (9HPT)**

The nine-hole peg test (9HPT) is used to measure finger dexterity.

- 1. Provide the participant with the 9 holes board.
  2. Inquire about possible conditions that could affect the ability to complete the test and record the chosen hand.
  3. The evaluator should perform the test in front of the participant, explaining the instructions.
  4. Instruct the participant to take the pegs from a container, one by one, and place them into the holes on the board, as quickly as possible, using only the hand being evaluated.
  5. Then, instruct the participant to remove the pegs from the holes, one by one, and replace them back into the container.
  6. The evaluator should start the stopwatch as soon as the patient touches the first peg.
  7. The evaluator should stop the stopwatch once the last peg is in the container.
  8. If a peg falls onto the table, the participant is asked to retrieve it and continue with the task.
  9. If a peg falls on the floor, the participant is asked to keep working on the task and the evaluator will retrieve it for the participant.
  10. After showing the methods to the participant, begin the first attempt.
